# A cross-sectional study on the quality of life of patients with type 2 diabetes attending a General Hospital in Lagos State

**DOI:** 10.11604/pamj.2025.51.92.45529

**Published:** 2025-08-13

**Authors:** Oyenike Oyeronke Ekekezie, Oluwadamilola Adetola Mogboyinola, Foluke Adenike Olatona

**Affiliations:** 1 Department of Senate and Governing Board Affairs, National Postgraduate Medical College of Nigeria, Lagos, Nigeria; 2 Department of Community Health and Primary Care, College of Medicine, University of Lagos, Lagos, Nigeria

**Keywords:** Quality of life, diabetic patients, treatment adherence, health literacy, general hospital, World Health Organization

## Abstract

**Introduction:**

diabetes mellitus is a progressive chronic disease that requires long-term management. This exerts a significant burden on patients, impacting their quality of life (QoL). The study aimed to assess the QoL and its determinants among patients with diabetes mellitus attending a general hospital in Lagos, Nigeria.

**Methods:**

a total of 306 patients diagnosed with diabetic mellitus were consecutively recruited from the clinic within the three-month study period. The World Health Organization quality of life (WHOQOL-BREF) tool was used for data collection. Multivariable logistic regression analysis was performed to explore the association between QoL and some clinical variables.

**Results:**

the majority of the respondents, 79.1% (n=242), were 50 years old and above, with a mean age of 53.7 + 0.6; 62.4% (n=191) females, 82.7% (n=253) married, and 56.2% (n=172) traders. The mean total QoL score was 52.9%. More than half, 55% (n= 163) of the patients had a good overall QoL score, while 76% (n= 233) perceived they had a good QoL. On multivariate regression analysis, QoL was significantly associated with normal fasting plasma glucose (aOR: 0.383, 95% CI: 0.191-0.768; p= 0.007); and adherence with therapy guidelines (aOR: 4.565, 95% CI: 2.375-8.774; p= 0.000).

**Conclusion:**

this study suggests that compliance with therapy guidelines was associated with the greatest odds of having good QoL. With our finding that higher levels of education were significantly associated with good QoL, it is recommended that health literacy become part of the management of patients with diabetes to enhance their QoL. There is a need to specifically target and improve the QoL of underweight patients with diabetes, as they are at a higher risk of having poor QoL, when compared with their overweight and obese counterparts that receive more attention.

## Introduction

The increasing burden of diabetes mellitus is a public health menace that places exhaustive demands on individuals, caregivers, and society at large [1]. The International Diabetes Federation (IDF) prevalence figures show that low- and middle-income nations have a higher burden of diabetes (367.8 million), compared with high-income countries (95.2 million) [2]. The prevalence rate of diabetes mellitus in Nigeria has been reported to vary from 2% to 12% across the country [3]. In 2018, a meta-analysis report estimated that 5.8% (about 6 million) of adult Nigerians were living with diabetes [4]. These figures are likely to be underestimations as the IDF reported that about 54% of diabetes cases in Nigeria were yet to be diagnosed, resulting in a significant strain on the healthcare system and an increase in the burden of diabetes complications and deaths [2,5,6]. Quality of Life (QoL) is how good or bad a person feels about their life [7]. The ability of medical care to prolong life, often at the expense of QoL, or to improve QoL without prolonging life has increased the importance of assessing QoL in health care [6]. Diabetes has a significant impact on the physical and psychological health of affected individuals, as well as on their social and economic well-being [8]. Diabetes has been demonstrated to have a strong negative impact on Health Related QoL (HRQoL), as its characteristic chronic high blood glucose level affects the microvasculature, resulting in complications [7,9-11]. If the demands of diabetes management do not fit how patients wish to live, they may choose to protect their QoL at the expense of adhering to therapy guidelines, making them more prone to complications, which negatively impacts their QoL [7,12].

Among patients with diabetes in the developing countries, a pattern of low to average QoL has been commonly reported. In Port Harcourt, Southern Nigeria, only 7.1% of the patients with diabetes had good overall QoL, and an Enugu study found that diabetes negatively impacted the HRQoL of the majority of the patients, when compared with age- and sex-matched patients without diabetes [13,14]. Persistent poor QoL among patients with diabetes can lead to gross dissatisfaction and mistrust for the healthcare system, which can make patients want to give up and not contribute their quota to the improvement of their diabetic condition. Poor QoL can affect the mental health of patients with diabetes and lead to depression, low self-worth, and suicidal thoughts [15]. Monitoring the health status of patients with diabetes without paying attention to the assessment of their QoL can mislead management decisions [7]. Assessment of their QoL will facilitate better management, assist in developing more appropriate interventions, and serve as a monitor for outcomes of diabetes care. It will also enhance the knowledge of the extent of the disease burden. This study aimed to assess the QoL and its determinants among patients with diabetes mellitus attending a general hospital in Lagos, Nigeria.

## Methods

**Study design and setting:** a hospital-based cross-sectional study was carried out between June and August 2023 in the diabetic clinic of the medical outpatient department of Ajeromi General Hospital, Ajegunle, located in Ajeromi-Ifelodun Local Government Area (LGA). It is one of the 24 hospitals under the management of the Lagos State Ministry of Health. Ajeromi-Ifelodun LGA is a sprawling urban slum, with residents engaged predominantly in petty trading and diverse commercial enterprise. The land size is 2,216 hectares, with about 2.2 million people reported to be resident in the LGA, comprising densely populated communities, and ranking as one of the most populated LGAs in the federation. Ajegunle has been described as ‘mini-Nigeria’ because of the large presence of almost every tribe/denomination and tongue that populate this sprawling land mass [16,17].

**Study population:** these were patients with a diagnosis of diabetes mellitus, attending the diabetic clinic of the medical outpatient department. To be eligible, they had to be aged 20 years and above, attending the clinic for at least one year prior to commencement of the study, and residing within Ajeromi-Ifelodun LGA. Patients with cognitive impairment and obvious disability that could affect the functions of the nervous system and independent self-care, and women with gestational diabetes were excluded from the study. The minimum sample size (n) was determined using Cochran´s formula [18].


$$\text{n}=\frac{Z^{2}pq}{d^{2}}$$

Z at 95% confidence level is 1.96; d is the margin of error at 5% (0.05), and p was estimated from the proportion of patients with diabetes with good overall QoL from a previous study among Nigerian adults with diabetes mellitus (20.7%) [19]. Accordingly, n=252; with 10% contingency, the minimum sample size was 277. Three hundred and six (306) eligible patients were consecutively recruited from the clinic within the three-month study period.

**Data collection:** this was carried out at the diabetic clinic which runs on three days a week: Mondays, Tuesdays and Thursdays, with an average of 30 patients per day. The patients attend once in two to three months, and at each visit, nursing assistants checked their blood pressure, fasting plasma glucose (FPG) and body mass index (BMI) before seeing the doctors. The questionnaire used to collect data had structured questions related to socio-demographic and clinical information, diabetic disease status, and the WHOQOL-BREF tool, which is a self-reporting questionnaire comprised of 24 items grouped into four domains of QoL: physical health, psychological health, social relationships and environment, and two items that measure overall QoL and general health. For each item, a 5 point-Likert-type scale was used [20]. The physical health domain was assessed using responses to seven items: pain and discomfort, dependence on medical treatment, energy and fatigue, mobility, sleep and rest, activities of daily living, and work capacity. The psychological health domain was assessed using responses to six items: positive feelings, spirituality and religious beliefs, thinking or memory and learning, bodily image and appearance, negative feelings, and self-esteem. The social relationship domain was assessed using responses to three items: personal relationships, social support, and sexual activities. The environment domain was assessed using responses to eight items: physical safety and security, physical environment, financial resources, opportunity for acquiring new information and skills, participation in and the opportunities for recreation and leisure activities, home environment, accessibility and quality of health and social care, and transportation. The raw scores for the domains of WHOQOL-BREF (4-20 score) were calculated by adding the values of single items, and these were transformed to the WHOQOL-100, with scores ranging from 0 to 100, for ease of comparison with other data sets. The mean score of each domain and the total score were calculated. The first two questions in WHOQOL-BREF were taken together for the analysis of Perceived QoL. Individuals with the total mean score of 50% and above were classified as having good perceived QoL and less than 50% as having poor perceived QoL. The questionnaire was pre-tested among 20 patients in General Hospital, Isolo which is located in a different LGA. The time taken to complete one interview was about 10 minutes during the pre-test.

### Definitions

**Dependent variable:** quality of Life was determined using the World Health Organization WHOQOL-BREF tool [20].

**Independent variables:** they include socio-demographic variables (age, gender, marital status, educational level, occupation and income). Clinical variables such as duration of diabetes, BMI, FPG, co-morbidities (hypertension, obesity, hyperlipidemia, heart disease, sleep disorders) and adherence to therapy were also evaluated.

**Blood pressure:** hypertension was defined as a blood pressure recording of ≥ 140/90 mmHg on more than 1 occasion or a documentation of treatment with anti-hypertensive medications [21].

**Fasting plasma glucose:** type 2 diabetes was defined as documentation of FPG ≥126 mg/dl (7.0 mmol/l) or 2 hours postprandial plasma glucose ≥ 200 mg/dl (11.1 mmol/l) for the first time in a patient, with or without classical symptoms of diabetes; or presentation for the first time with symptoms of hyperglycemia and a documented random plasma glucose ≥ 200 mg/dl (11.1 mmol/l) [22].

**Body mass index:** BMI was classified as underweight (BMI <18.5), normal (BMI 18.5-24.9), overweight (BMI 25.0-29.9), obesity (BMI ≥ 30) [23].

**Statistical analysis:** data collected were analyzed using the Statistical Package for Social Sciences (SPSS version 29) by IBM Corporation, Armonk, New York, United States. Descriptive analysis was carried out for socio-demographic variables such as age, sex, educational attainment and income; as well as for clinical variables such as duration of diabetes, co-morbidities, complications and adherence to therapy. To explore the association between QoL and some clinical variables, a univariate logistic regression analysis was first conducted with QoL (good or poor) as the dependent variable and the clinical variables as the predictor. This was followed by a multivariate logistic regression analysis to adjust for potential confounders, including age, sex, educational attainment and income. A P value of <.05 was considered statistically significant. Interpretation of results focused on the magnitude and direction of the adjusted beta coefficients, their 95% confidence intervals, and P values, to determine the strength and significance of associations between QoL and the clinical variables while controlling for other factors.

**Ethical considerations:** ethical approval was obtained from the Health Research and Ethics committee of the Lagos University Teaching Hospital before the commencement of the study. (The approval number is ADM/DSCDT/HREC/APP/5843). Informed written consent was obtained from each participant and confidentiality was maintained throughout the study.

## Results

**General characteristics of the study population:** a total of 306 participants, made up of 62.4% (n= 191) females, 82.7% (n= 253) married, and 56.2% (n= 172) traders, diagnosed of diabetes mellitus, who met the inclusion criteria, were recruited into the study. The mean age of the participants was 53.7 + 0.6 years. Sixty-three percent (n= 193) had their blood glucose level under control, 61% (n= 188) had been managing diabetes for 6-10 years, and 63% (n= 193) had diabetic complications. Over half, 52.3% (n= 160) were of normal weight, and about a quarter, 28.8% (n=88) were overweight. The most prevalent complication was neuropathy, 32.0% (n= 98), and hypertension was the most prevalent comorbidity, 57.8% (n= 177).

**Figure 1:**
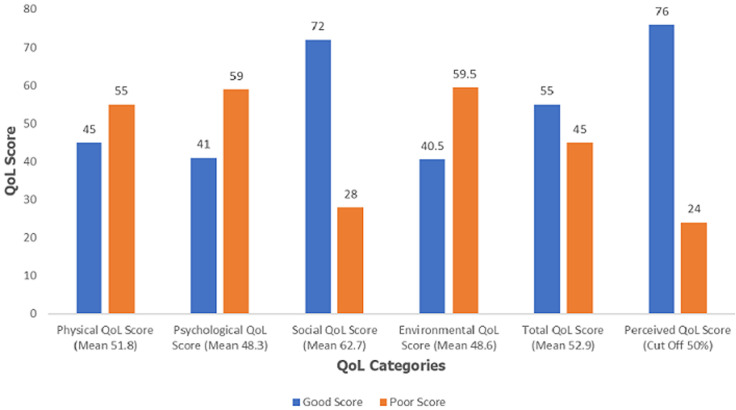
quality of life categories

**Quality of life (QoL) scores and categories:** based on the WHOQOL-BREF (4-20 score), the mean total QoL score was 13.2 ± 1.9. The domain scores were transformed to the WHOQOL-100 (0-100 score); based on which the mean total QoL score was 52.9 ± 10.3 (Table 1). Fifty-five percent of the patients (n= 163) had overall good QoL score, whereas 76% (n= 233) perceived they had good QoL score. The social domain had the highest proportion of respondents, 72% (n= 220) with good QoL score. The psychological and environmental domains both had the lowest proportion of respondents, about 41% (n= 125) with good QoL score, and also, the highest proportion 59.5% (n= 182) with poor QoL score (Figure 1).

**Table 1:** quality of life (QoL) scores

Domains	No	Minimum	Maximum	Mean	Standard deviation
**Quality of life scores based on WHOQOL-BREF**					
Physical QoL	306	7.4	18.9	12.3	1.7
Psychological QoL	306	6.7	18.7	11.7	1.7
Social QoL	306	8	20	14	2.5
Environmental QoL	306	7.5	20	11.8	1.9
Total QoL score	306	8.5	19.4	13.2	1.9
**Quality of life scores based on WHOQOL-100**					
Physical QoL	306	21.6	93.2	51.8	10.9
Psychological QoL	306	17	92	48.3	10.3
Social QoL	306	25	100	62.7	15.3
Environmental QoL	306	22	100	48.6	11.8
Mean total QoL score	306	29.3	92.6	52.9	10.3

**QOL:** quality of life; WHOQOL: World Health Organization quality of life

**Factors associated with QoL:** fasting plasma glucose, BMI, co-morbidities and adherence to therapy were significantly associated with QoL in the univariate regression analysis (Table 2). A multivariate regression model was constructed to assess the independent association between QoL and the clinical factors, while adjusting for potential confounders which included age, sex, marital status, educational attainment, occupation and income (Table 2). There was a significant positive association between normal FPG and QoL (aOR: 0.383, 95% CI: 0.191-0.768; p= 0.007). This indicates that compared to the reference group (patients with high FPG), patients with normal FPG had 62% lower odds of having poor QoL (Table 2). Compared to the reference group (patients in the underweight category), those in the overweight category had a significant positive association with QoL (aOR: 0.145, 95% CI: 0.043-0.485; p = 0.002), suggesting being overweight confers 86% lower odds of having poor QoL, compared with being underweight (Table 2). By the same token, non-compliance with therapy guidelines was significantly associated with QoL (aOR: 4.565, 95% CI: 2.375-8.774; p= 0.000), indicating non-compliance resulted in 4.5 times greater odds of having poor QoL compared with those who complied (reference group) (Table 2). Some co-morbidities (hyperlipidemia and sleep disorders) showed significant positive association with QoL (aOR: 0.025, 95% CI: 0.002-0.285; p= 0.003) and (aOR: 0.029, 95% CI: 0.002-0.464; p= 0.012) respectively. This suggests that patients with hyperlipidemia and sleep disorders had 97.5% and 97% lower odds of having poor QoL respectively, when compared with patients with heart disease (reference group) (Table 2). Being male (aOR: 1.994; 95% CI: 1.101-3.612; p= 0.023), widowed (aOR: 5.225, 95% CI: 1.253-21.783; p= 0.023), and an Nigerian Certificate in Education/Ordinary National Diploma (NCE/OND) holder (aOR: 2.922, 95% CI: 1.467-5.820; p= 0.002) were associated with greater odds (2 times, 5 times, and 3 times respectively) of having poor QoL, compared with being female, married, or holding a Bachelor´s degree (Table 2).

**Table 2:** factors associated with quality of life (n=306)

	Quality of Life			
Categories	Unadjusted ORs (95% CI)	P-value	Adjusted ORs (95% CI)	P-value
**Fasting plasma glucose**				
70-99mg/dl (low)	829568162.545 (0.00)	1.000	612952377.412 (0.000)	1.000
100-125mg/dl (normal)	0.243 (0.148-0.398)	0.000	0.383 (0.191-0.768)	0.007
**Body mass index**				
18.5 - 25 (normal weight)	0.344 (0.142-0.833)	0.018	0.589 (0.182-1.902)	0.376
>25 - 30 (overweight)	0.196 (0.077-0.503)	0.001	0.145 (0.043-0.485)	0.002
>30 (Obese)	0.583 (0.196-1.737)	0.333		
**Adherence to therapy**				
Non-compliance	4.716 (2.868-7.755)	0.000	4.565 (2.375-8.774)	0.000
**Co-morbidities**				
Hypertension	0.221 (0.026-1.878)	0.167	0.346 (0.034-3.532)	0.370
Hyperlipidemia	0.034 (0.004-0.314)	0.003	0.025 (0.002-0.285)	0.003
Obesity	0.231 (0.025-2.155)	0.198	0.125 (0.008-1.844)	0.130
Sleep disorders	0.024 (0.002-0.315)	0.005	0.029 (0.002-0.464)	0.012
None	0.000 (0.000)	0.998	0.000 (0.000)	0.998
**Sex**				
Male	1.916 (1.199-3.061)	0.007	1.994 (1.101-3.612)	0.023
**Marital**				
Unmarried	0.524 (0.248-1.107)	0.090	0.783 (0.343-1.786)	0.561
**Education**				
SSCE	1.013 (0.513-2.002)	0.971	1.355 (0.635-2.890)	0.432
NCE/OND	2.244 (1.251-4.025)	0.007	2.922 (1.467-5.820)	0.002
Masters	0.147 (0.018-1.202)	0.074	0.109 (0.012-1.017)	0.052
Others	0.853 (0.349-2.087)	0.728	0.998 (0.353-2.821)	0.997

ORs: odds ratios; SSCE: Secondary school certificate examination; NCE/OND: Nigerian Certificate in Education/Ordinary National Diploma

## Discussion

The objective of this study was to assess the QoL and its determinants among patients with diabetes mellitus attending a general hospital in Lagos, Nigeria. In this study, the social domain had the highest proportion of respondents with good QoL score (about three-quarters of them); while the psychological and environmental domains both had the lowest proportion of respondents with good QoL score, and the highest proportion with poor QoL score (about two-fifths of them). The multivariate regression model suggested that patients with normal FPG had 62% lower odds of having poor QoL compared with those with high FPG; being overweight conferred 86% lower odds of having poor QoL, compared with being underweight; and non-compliance with therapy guidelines resulted in 4.5 times greater odds of having poor QoL compared with those who complied. The model also showed that the odds of having poor QoL was two times higher in male participants compared to females; five times higher in widowed participants compared to married; and three times higher in NCE/OND holders compared to those having a Bachelors´ degree. The QoL scores found in this study are not unexpected among patients residing in an overpopulated sprawling urban slum, which is one of the most populous LGA in Nigeria, albeit located in the smallest State in the federation. A lot of pressure will be exerted on the social, economic and health infrastructure the State and LGA are able to provide. The implication is that the socio-economic responsibilities borne by residents will be overwhelming, more so when the health and economic burden of diabetes are added [14,17].

The social domain however, stood out with almost three-quarters of the participants having good QoL. The facets measured in the WHOQOL-BREF instrument pertaining to social QoL are relationships, social support and sex. Nigerians are social people, they value family and friendships, especially within the lower socio-economic classes, and there is usually an abundant supply of social support. This finding is consistent with that among patients with diabetes in the rural area of Neyshabur, North Eastern Iran, where socio-economic, physical, psychological and environmental challenges also weigh heavily on the people. The domain with the highest QoL score of 54.16 was also the social domain, while the lowest was the psychological domain (48.39) [24]. Perceived QoL is an individual´s overall perception of his own QoL and health. Seventy-five percent of the respondents believed that their quality of life is good, despite the socio-economic challenges; physical, psychological and environmental stressors they face, which reflected in their mean (measured) total QoL score of 52.9. The most essential feature of assessing QoL is the individual´s subjective evaluation of their own QoL (perceived), and not what others imagine it to be [7]. Nigerians have commonly been referred to as ‘happy people’ despite the harsh realities of the times. The QoL score of the social domain towering above the other suboptimal domains probably influenced their perception. This is in agreement with findings from a study in rural South India, where the mean total QoL score was 58.03, and 72% perceived their QoL was good [7]. The domain scores reported in the rural South India study were low, in keeping with people residing in a community with low education, low standards of living and poor socio-economic status, but with an exception: 85% had good environmental QoL scores. The availability in the district of good roads, transportation facilities, as well as access to good public health infrastructure could have influenced their environmental QoL scores, despite their individual living condition.

Among the diabetics studied, having controlled FPG, adhering to therapy, having a BMI in the overweight range, being female, currently married, and having tertiary education, were significantly associated with better QoL, compared to their counterparts. This is in agreement with some of the factors associated with good QoL reported among 43% of patients with diabetes in the Republic of Benin: education, marital status, family history, social support, and diabetic complications [25]. In Vellore, rural South India, factors influencing QoL were consistent with those found in this study, except males rather than females having better QoL [7]. Another study on the assessment of QoL and its determinants among patients with diabetes using the same WHOQOL-BREF tool in Bangladesh found that, education, employment status, income, and comorbid conditions such as hypertension, obesity, high serum cholesterol level, and microvascular complications affect QoL [26]. Findings in this study suggest that being underweight confers significantly greater odds of having poor QoL compared with being overweight, as well as compared with obese patients, though not significant. Almost three-quarters of the underweight patients with diabetes had poor QoL. Greater attention should be directed to underweight patients with diabetes to improve treatment outcomes, as many more studies have highlighted poor QoL among obese patients with diabetes [27-29].

The patients who adhered to therapy guidelines in our study had the greatest odds of having good QoL. Adherence to treatment guidelines results in controlled FPG levels, which translates to a reduction in the risk of developing complications, which would have negatively impacted the QoL of patients. This is consistent with another study across four cities in Alagoas, Brazil, that found an association between the social domain of QoL and treatment adherence [30]. Similarly, a systematic review of six studies found that the combination of the psychological and social domains of QoL had the greatest impact on treatment adherence [31]. Other studies associating adherence and controlled FPG with good QoL were found in India and Indonesia [32,33]. Our finding that higher levels of education were significantly associated with good QoL is consistent with findings documented among patients with diabetes in Indonesia and Iran [34,35]. Education is associated with better knowledge of diabetes and its management, which assists the patients in being self-efficacious and communicate better while taking care of their health, promoting adherence [36]. Poor literacy of patients with diabetes has been linked to various complications, including hypoglycemia, indirectly affecting the QoL of the patients [36,37]. Diabetes specific instruments to measure QoL, such as Diabetes Quality of Life (DQOL), and Diabetes Health Related Quality of Life (DHRQOL) could not be used in this study because locally validated versions of these tools are not yet available. However, the WHOQOL-BREF tool which is used for measuring general QoL gives a good estimate. A multi-center study and a larger sample size are necessary to more comprehensively examine the factors that affect the QoL of patients with diabetes.

## Conclusion

This study suggests that compliance with therapy guidelines was associated with the greatest odds of having good QoL. With our finding that higher levels of education were significantly associated with good QoL, it is recommended that health literacy become part of the management of patients with diabetes to enhance their QoL. There is a need to specifically target and improve the QoL of underweight patients with diabetes, as they are at a higher risk of having poor QoL, when compared with their overweight and obese counterparts, that receive more attention. The high social QoL score probably influenced the patients´ perception of their QoL to be much higher than the measured QoL. Social amenities, infrastructure and programs can be developed to have similar effects on the quality of life of patients with diabetes and other chronic diseases.

### What is known about this topic

- A pattern of low to average quality of life has been commonly reported among patients with diabetes in the developing countries;
- Adherence to therapy and controlled fasting plasma glucose have been associated with good quality of life;
- Greater odds of poor quality of life among obese patients with diabetes.

### What this study adds

- Findings in this study suggest being underweight confers significantly greater odds of having poor quality of life compared with being overweight and obese;
- Compliance with therapy guidelines was associated with the greatest odds of having good quality of life.

## References

[1] Kałucka S, Kaleta D, Makowiec-Dabrowska T (2019). Prevalence of Dietary Behavior and Determinants of Quality of Diet among Beneficiaries of Government Welfare Assistance in Poland. Int J Environ Res Public Health.

[2] Cho NH, Shaw JE, Karuranga S, Huang Y, da Rocha Fernandes JD, Ohlrogge AW (2018). IDF Diabetes Atlas: Global estimates of diabetes prevalence for 2017 and projections for 2045. Diabetes Res Clin Pract.

[3] Nyenwe EA, Odia OJ, Ihekwaba AE, Ojule A, Babatunde S (2003). Type 2 diabetes in adult Nigerians: as study of its prevalence and risk factors in Port Harcourt, Nigeria. Diabetes Res Clin Pract.

[4] Uloko AE, Musa BM, Ramalan MA, Gezawa ID, Puepet FH, Uloko AT (2018). Prevalence and risk factors for diabetes mellitus in Nigeria: a systematic review and meta-analysis. Diabetes Ther.

[5] National Library of Medicine IDF Diabetes Atlas: Global Picture.

[6] The International Diabetes Federation (IDF) IDF Diabetes Atlas: diabetes around the world in 2021.

[7] Manjunath K, Christopher P, Gopichandran V, Rakesh PS, George K, Prasad JH (2014). Quality of life of a patient with type 2 diabetes: a cross-sectional study in rural South India. J Family Med Prim Care.

[8] Mapa-Tassou C, Katte JC, Mba Maadjhou C, Mbanya JC (2019). Economic impact of diabetes in Africa. Curr Diab Rep.

[9] vanNetten JJ, Price PE, Lavery LA, Monteiro-Soares M, Rasmussen A, Jubiz Y (2016). Prevention of foot ulcers in the at-risk patient with diabetes: a systematic review. Diabetes Metab Res Rev.

[10] Faselis C, Katsimardou A, Imprialos K, Deligkaris P, Kallistratos M, Dimitriadis K (2020). Microvascular complications of type 2 diabetes mellitus. Curr Vasc Pharmacol.

[11] Nagpal J, Kumar A, Kakar S, Bhartia A (2010). The development of Quality of Life Instrument for Indian Diabetes patients (QOLID): a validation and reliability study in middle-and higher-income groups. J Assoc Physicians India.

[12] Souza MA de, Freitas RWJF de, Lima LS de, Santos MA dos, Zanetti ML, Damasceno MMC (2019). Health-related quality of life of adolescents with type 1 diabetes mellitus. Rev Lat Am Enfermagem.

[13] Nnachi C, Alabere I, Asuquo EAE, Oti IK (2022). Quality of life of type 2 patients with diabetes attending a tertiary hospital in South-South Nigeria: Quality of life of type 2 diabetic. Nigerian Journal of Medicine.

[14] Nwatu CB, Onyekonwu CL, Unaogu NN, Ijeoma UN, Onyeka TC, Onwuekwe IO (2019). Health-related quality of life in Nigerians with complicated diabetes mellitus-a study from Enugu, South East Nigeria. Niger J Med.

[15] Robinson S, Kissane DW, Brooker J, Hempton C, Burney S (2017). The relationship between poor quality of life and desire to hasten death: A multiple mediation model examining the contributions of depression, demoralization, loss of Control, and low self-worth. J Pain Symptom Manage.

[16] City Population Nigeria: Administrative Division (States and Local Government Areas).

[17] Ajeromi-Ifelodun Local Government (2023). About Ajeromi-Ifelodun LGA.

[18] Cochran WG (1977). Sampling Techniques.

[19] Issa BA, Baiyewu O (2006). Quality of life of patients with diabetes mellitus in a Nigerian teaching hospital. East Asian Arch Psychiatry.

[20] World Health Organization (2004). The World Health Organization Quality of Life (WHOQOL-BREF). WHO.

[21] World Health Organization (2004). A global brief on hypertension: silent killer, global public health crisis: World Health Day 2013. WHO.

[22] American Diabetes Association (2016). Standards of Medical Care in Diabetes-2016. Diabetes Care.

[23] World Health Organization (1995). Physical status: the use and interpretation of anthropometry. WHO.

[24] Gholami A, Azini M, Borji A, Shirazi F, Sharafi Z, Zarei E (2013). Quality of life in patients with type 2 diabetes: Application of WHOQOL-BREF scale. Shiraz E-Med J.

[25] Alaofe H, AmoussaHounkpatin W, Djrolo F, Ehiri J, Rosales C (2022). Factors associated with quality of life in patients with type 2 diabetes of south Benin: A cross-sectional study. Int J Environ Res Public Health.

[26] Amin MF, Bhowmik B, Rouf R, Khan MI, Tasnim SA, Afsana F (2022). Assessment of quality of life and its determinants in type-2 diabetes patients using the WHOQOL-BREF instrument in Bangladesh. BMC Endocr Disord.

[27] Rozjabek H, Fastenau J, LaPrade A, Sternbach N (2020). Adult obesity and health-related quality of life, patient activation, work productivity, and weight loss behaviours in the United States. Diabetes Metab Syndr Obes.

[28] Stephenson J, Smith CM, Kearns B, Haywood A, Bissell P (2021). The association between obesity and quality of life: a retrospective analysis of a large-scale population-based cohort study. BMC Public Health.

[29] Hlatky MA, Chung SC, Escobedo J, Hillegass WB, Melsop K, Rogers W (2010). The effect of obesity on quality of life in patients with diabetes and coronary artery disease. Am Heart J.

[30] Alves de Farias MSJ, Lumack do Monte Agra CC, Alves de Araujo LK, Correia DS, Cavalcante JC (2014). Treatment adherence and life quality of patients with diabetes assisted in the primary care division. Rev Soc Bras Cl??n Med.

[31] Gusmai L de F, Novato T de S, Nogueira L de S (2015). The influence of quality of life in treatment adherence of patients with diabetes: a systematic review. Rev Esc Enferm USP.

[32] Mishra R, Sharma SK, Verma R, Kangra P, Dahiya P, Kumari P (2021). Medication adherence and quality of life among type-2 diabetes mellitus patients in India. World J Diabetes.

[33] Perwitasari DA, Urbayatun S (2016). Treatment adherence and quality of life in diabetes mellitus patients in Indonesia. Sage Open.

[34] Akrom A, Anggitasari W (2019). Adherence and quality of life among patients with diabetes with hypertension. Int J Publ Health Sci.

[35] Zare F, Ameri H, Madadizadeh F, Reza Aghaei M (2020). Health-related quality of life and its associated factors in patients with type 2 diabetes mellitus. SAGE Open Med.

[36] Bailey SC, Brega AG, Crutchfield TM, Elasy T, Herr H, Kaphingst K (2014). Update on health literacy and diabetes. Diabetes Educ.

[37] Stormacq C, Van den Broucke S, Wosinski J (2019). Does health literacy mediate the relationship between socioeconomic status and health disparities?. Integrative review. Health Promot Int.

